# Preparation of Lignin-Based Magnetic Adsorbent From Kraft Lignin for Adsorbing the Congo Red

**DOI:** 10.3389/fbioe.2021.691528

**Published:** 2021-06-07

**Authors:** Lingyan Fang, Hao Wu, Yuxuan Shi, Yuheng Tao, Qiang Yong

**Affiliations:** ^1^Jiangsu Co-Innovation Center for Efficient Processing and Utilization of Forest Resources, College of Chemical Engineering, Nanjing Forestry University, Nanjing, China; ^2^Department of Biomedical Engineering, School of Biomedical Engineering and Informatics, Nanjing Medical University, Nanjing, China

**Keywords:** kraft lignin, Mannich reaction, Congo red, magnetic adsorbent, preparation

## Abstract

The utilization of lignin from different lignocellulosic biomass is the hot topic for the biorefinery of biomass. In this paper, magnetic lignin nanoparticles (MLN) were prepared by kraft lignin from bamboo residue and Fe_3_O_4_ with different ratios via Mannich reaction. The surface morphology and structure of magnetic lignin were characterized and analyzed by X-ray powder diffraction, Fourier transform infrared spectroscopy, and transmission electron microscopy, which confirmed that the MLN were successfully prepared. The performance of MLN adsorbents was evaluated by adsorbing Congo red solution at different initial concentrations and contact times. The results showed that Fe_3_O_4_@lignin (1:0.5) had the best adsorption effect on Congo red solution. When the concentration of Congo red reached 0.6 g/L, Fe_3_O_4_@lignin (1:0.5) had the best adsorption effect on Congo red, reaching 95.5% in only 30 min. As lignin is modified by Fe_3_O_4_, it can be recovered by magnetic substances after adsorption and has good reuse performance. The results of adsorption kinetics and adsorption isotherm showed that except for the adsorption process of Fe_3_O_4_@lignin (1:0.5), which is consistent with the chemical adsorption of the multimolecular layer, the adsorption process of other adsorbents is in accordance with the chemical adsorption of the monomolecular layer. In terms of environmental protection and adsorption efficiency, and MLN has become an ideal adsorbent for Congo red dyes due to its simple preparation, superior performance, and convenient recovery.

## Introduction

Due to the impact of environmental issues of dye wastewater from printing and dyeing mill on public health, it has become a global concern (Liu J. et al., 2018). Most of these dyes are soluble in water, complex in composition, and high in organic pollutants (Liu L. et al., 2018). Most of them are difficult to biodegrade and even cause “Triple induction” (carcinogenic, teratogenic, mutagenic) (Konicki et al., 2018; Sham and Notley, 2018). Therefore, the dye wastewater must be properly treated and protected, otherwise it will cause harm to aquatic species and the environment (Ghaedi et al., 2012; Gu et al., 2021). Congo red is a typical biphenyl amine azo dye, which will produce toxic substances in an anaerobic environment and easily accessible to water bodies during industrial production and use (Zhao et al., 2018). It is one of the representative pollutants in printing and dyeing wastewater. At present, the treatment methods of dye wastewater include radiation method, magnetic separation method, an adsorption method, membrane separation method, and photocatalytic oxidation method (Holkar et al., 2016; Cui et al., 2018; Wang et al., 2018b). Adsorption method is a common method for wastewater treatment because it is efficient, simple, recyclable, produces less secondary pollution, and does not change the structure of pollutants in dye wastewater (Yang et al., 2019).

In recent years, several adsorbents such as activated carbon, zeolite, ion exchange materials, and bentonite have been widely used in dye adsorption. However, due to the problems of difficult recovery, difficulty in regeneration and reuse, high price, and low adsorption efficiency of these adsorbents, if these adsorbents are used for large-scale treatment of dye wastewater adsorption, the cost is relatively expensive (Hassan and Carr, 2018).

As a renewable biomass material, lignin is one of the main components in nature plants, with a wide range of sources (Klapiszewski et al., 2017a; Pei et al., 2020; Zheng et al., 2021). In industry, lignin is mainly separated and extracted from black liquor discharged during the pulping and papermaking process, and can be processed into various functional materials with high value and applied (Kai et al., 2016; Dong et al., 2020a). Lignin contains a large number of functional groups such as benzene ring, hydroxyl group, carbonyl group, carboxyl group, methoxy group, and unsaturated bond (Gall et al., 2017; Dong et al., 2020b). It has the ability of ion exchange and adsorption, which is more advantageous than other adsorbents such as activated carbon, macromolecule resin, and minerals, and has attracted extensive attention (Upton and Kasko, 2016). However, lignin is difficult to separate and recover, and its application is greatly restricted (Humpert et al., 2016). It can be solved by combining with magnetic materials to produce magnetic lignin nano-materials. The magnetic components are mainly nickel, iron, cobalt, iron, and alloy oxides such as γ-Fe_2_O_3_ and Fe_3_O_4_. Fe_3_O_4_ is widely used as the magnetic component of magnetic polymer materials due to its advantages of simple preparation process, stable performance, and low toxicity (Siyasukh et al., 2018; Wang et al., 2018a; Lou et al., 2020, 2021). MLN, such as Fe_3_O_4_@lignin, can be prepared by Mannich reaction, which is a condensation reaction between amine compounds and aldehydes and containing active hydrogen atoms in lignin (Wang B. et al., 2018). The Mannich reaction usually involves the formation of *N*-hydroxymethyl amines by the reaction of the amine group with the aldehyde group, and condensation of the hydrogen atoms by the substitution of the amine group, namely, the amine methylation reaction (Jiao et al., 2019).

This reaction is particularly useful for the synthesis of β-aminocarbonyl derivatives (Kobayashi et al., 2011). In the Mannich reaction, the crosslinking agent between fatty amine and lignin consists of an aldehyde group, and formaldehyde is currently the most widely used aldehyde (Gao et al., 2020). Because the Mannich reaction is simple, effective, and without by-products, it is widely used in the synthesis of new materials (Guo et al., 2020). Luo et al. (2017) recovered lignin from black liquor, modified the lignin by Mannich reaction with triethylenetetramine (TETA), and then chelated iron to the aminated lignin to obtain a highly efficient phosphate adsorbent. The magnetic lignin prepared can be simply recovered by using a magnetic substance. Therefore, lignin can be fully utilized, and lignin can be recycled and reused through magnetic properties, thereby improving the economic value of lignin (Calvo-Flores and Dobado, 2010). In the current research, few people load lignin on magnetic nanoparticles of Fe_3_O_4_ and use the MLN for dye adsorption, which is also the starting point of our work.

In this study, the magnetic Fe_3_O_4_ with good performance was prepared by co-precipitation method, and then it reacted with ethylsilicate (TEOS) and (3-aminopropyl) triethoxysilane (APTES) to obtain the aminated Fe_3_O_4_. Then, the aminated Fe_3_O_4_ was reacted with lignin to obtain a green recyclable MLN adsorbent. The surface morphology and phase composition of magnetic lignin were characterized and analyzed by X-ray powder diffraction (XRD), Fourier transform infrared spectroscopy (FT-IR), transmission electron microscopy (TEM), and apply it to the removal of Congo red. In addition, the adsorption kinetics and adsorption isotherm of MLN for adsorbing the Congo red were also evaluated.

## Materials and Methods

### Materials

Kraft lignin was obtained from black liquor of bamboo residues via acid precipitation. The textile dye Congo red was purchased from China National Pharmaceutical Holding Chemical Reagent Co., Ltd. and was used without further purification. All other chemicals were also used without any other purification.

### Preparation of Fe_3_O_4_

Fe_3_O_4_ was prepared by the co-precipitation method, based on the principle of Fe^2+^+2Fe^3+^+8OH^–^ = Fe_3_O_4_+4H_2_O. Specifically, 6.1 g FeCl_3_⋅6H_2_O and 4.2 g FeSO_4_⋅7H_2_O were dissolved in 100 ml of deionized water, added into a 250-ml three-necked flask. Then the solution was heated to 85°C in an oil bath under an argon atmosphere and mechanically stirred at 300 rpm. When at 70°C, 10 ml ammonia water was added and continued to heat for 1 h at 85°C. After the reaction was finished, the black precipitate was separated from the reaction medium by an external magnetic field and ultrasonically washed three times with deionized water until the pH is neutral, to obtain Fe_3_O_4_ particles.

### Preparation of Fe_3_O_4_ @lignin Composites

Fe_3_O_4_ nanoparticles prepared by the aforementioned method were dispersed in 150 ml of ethanol/water (4:1, v/v) solution, and then the mixture was dispersed uniformly in ultrasonic to form magnetic fluid and added into a 250-ml three-necked flask. Then 5 ml of tetraethyl orthosilicate (TEOS) was added, and ammonia water was used to adjust the pH to 9.0. Then the solution was heated to 45°C for 16 h in an oil bath under an argon atmosphere and mechanically stirred at 300 rpm. After 16 h, different proportions of lignin solution (1:0.5, 1:1, 1:2, 1:3), 7 ml formaldehyde, and 10 ml 3-aminopropyltriethoxysilane (APTES) were added to the three-necked flask, then heated to 60°C for 6 h. To prepare the lignin solution, 1.37, 2.74, 5.48, and 8.22 g lignin in black liquor were dissolved in 20 ml ammonia solution, which was proposed based on the amount of Fe_3_O_4_ to obtain Fe_3_O_4_@lignin with ratios of 1:0.5, 1:1, 1:2, and 1:3. After the reaction was finished, the mixture was washed with ethanol several times until the pH is neutral. The precipitated solid (MLN) was obtained by frozen drying. The obtained MLN with different ratios of 1:0.5, 1:1, 1:2, and 1:3 for Fe_3_O_4_ and lignin were termed as Fe_3_O_4_@lignin (1:0.5), Fe_3_O_4_@lignin (1:1), Fe_3_O_4_@lignin (1:2), and Fe_3_O_4_@lignin (1:3).

### Characterization of MLN

The composition of the sample was analyzed by model Rigaku Ultima IV X-ray powder diffraction. The chemical bond structure of the sample was determined using an infrared spectrometer (VERTEX 80V; Bruker, Germany). The internal morphology and structure of the sample was observed by JEM-2100 transmission electron microscope. The magnetic properties of the samples were measured by a Lake Shore 7404 vibrating sample magnetometer.

### Determination of Iron Content

Determination of iron content in Fe_3_O_4_@lignin composite was performed by the phenanthroline method, which is one of the common methods used for the determination of iron content. The key to determining the iron content by the phenanthroline method is to disperse the nano-iron oxide particles under acidic conditions to dissolve them into Fe^2+^/Fe^3+^, and under the action of a reducing agent, all the iron ions in the solution are reduced to Fe^2+^. At this time, the orange-red complex formed in the solution is a complex of phenanthroline and Fe^2+^. According to the literature, the maximum absorption peak of the orange-red complex is obtained at 510 nm. The absorbance of the spot is measured by an ultraviolet–visible spectrophotometer, and the iron content of the sample to be tested can be obtained by comparing the drawn standard curve and the curve equation.

### Adsorption Experiment

The effect of magnetic lignin on Congo red concentration adsorption and the effect of magnetic lignin on Congo red adsorption time were determined. Congo red solution was prepared with concentration ranging from 0.01 to 0.8 g/L. Then 20 ml Congo red solution was added into a 50-ml Erlenmeyer flask as well as the addition of 0.05 g magnetic lignin, subsequently reacted at 30°C and 150 rpm for 4 h. The absorption value of supernate was measured at λ_*max*_ of 496 nm. Further, the adsorption rate and the absorption capacity were calculated.

The effect of magnetic lignin on adsorption time of Congo red was determined. Forty milliliters of 0.6 g/L Congo red solution was added with 0.1 g of magnetic lignin and shaken at 150 rpm for 5 h. The absorbance value of supernate was measured at *λ*_*max*_ of 496 nm to determine the concentration of Congo red. Further, the adsorption rate and the absorption capacity were calculated.

In all experiments, the equilibrium adsorption amount q_*e*_ (mg/g) was determined by the mass balance of the dye:

(1) $$q_{e}=\frac{\left(C_{o}-C_{e}\right)\times V}{\text{m}}$$

The initial concentration and adsorption equilibrium concentration of the dyes were C_*o*_ (mg/L) and C_*e*_ (mg/L), respectively. V (ml) is dye solution’s volume and m (g) is adsorbent’ s amount.

### Adsorption Kinetics

To study the control mechanism of the adsorption process, such as mass transfer or chemical reaction, the pseudo-first-order, pseudo-second-order dynamic models and the intraparticle diffusion model given in Eqs. 2–4 were used respectively.

(2) $$\text{log}\,q_{e}-q_{t}=\text{log}\,q_{e}-\frac{k_{1}}{2.303}\,t$$

(3) $$\frac{t}{q_{t}}=\frac{1}{q_{e}^{2}\,k_{2}}+\frac{t}{q_{e}}$$

(4) $$q_{t}=k_{i}\,t^{1/2}+C$$

q_*t*_ (mg/g) is the adsorption capacity at t (min); q_*e*_ (mg/g) is the adsorption capacity when equilibrium is reached, t is the adsorption time, and C is a constant related to the boundary layer thickness; k_1_ (min^–1^) and k_2_ (g mg^–1^ min^–1^) are pseudo-first-order and pseudo-second-order kinetic adsorption rate constants, respectively, and k_*i*_ (mg/g⋅min^1/2^) is the intracellular diffusion rate constant. q_*e*_ and k_1_, k_2_, and k_*i*_ can be determined from the experimental data by equations.

### Adsorption Isotherm

The adsorption equation of the Langmuir model is shown in Eqs. 5 and 6:

(5) $$\frac{C_{e}}{q_{e}}=\frac{1}{q_{m}\,b}+\frac{C_{e}}{q_{m}}$$

(6) $$R_{L}=\frac{\text{a}}{\left(1+b\,C_{o}\right)}$$

q_*e*_: Adsorption mass (mg/g) during adsorption equilibrium of Congo red solution

C_*e*_: equilibrium concentration of Congo red solution (mg/L)

q_*m*_: maximum adsorption value of adsorbent (mg/g)

b: Langmuir adsorption constant (L/mg)

Adsorption equation of Freundlich model is shown in Eq. 7:

(7) $$\ln\left(q_{e}\right)=\ln\left(K_{f}\right)+\frac{1}{n}\,\ln\left(C_{e}\right)$$

q_*e*_: Adsorption mass (mg/g) during adsorption equilibrium of Congo red solution

C_*e*_: equilibrium concentration of Congo red solution (mg/L)

K_*f*_: Freundlich equilibrium constant, roughly indicating the adsorption capacity of the adsorbent (mg/g)

$\frac{1}{n}$: Heterogeneity factor, related to adsorption strength. When 0 < l/n < l, the adsorption is advantageous; if 1/n = 1, the adsorption is linear, there is no interaction between adsorbates; when l/n > l, the adsorption is negative (Wang et al., 2018b).

## Results and Discussion

### Characterization of Adsorbents

**FIGURE 1:**
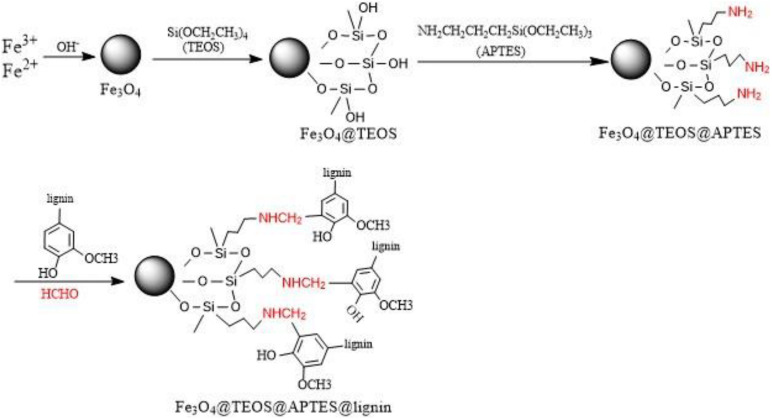
Mannich reaction synthesis diagram. TEOS, tetraethyl orthosilicate; APTES, 3-aminopropyltriethoxysilane.

In this paper, through the Mannich reaction, the magnetic Fe_3_O_4_ reacted with TEOS and APTES in turn, and finally reacted with lignin to obtain MLN. The specific mechanism is shown in Figure 1. Characterization analysis was performed on Fe_3_O_4_@lignin to understand the structural characteristics of the samples.

**FIGURE 2:**
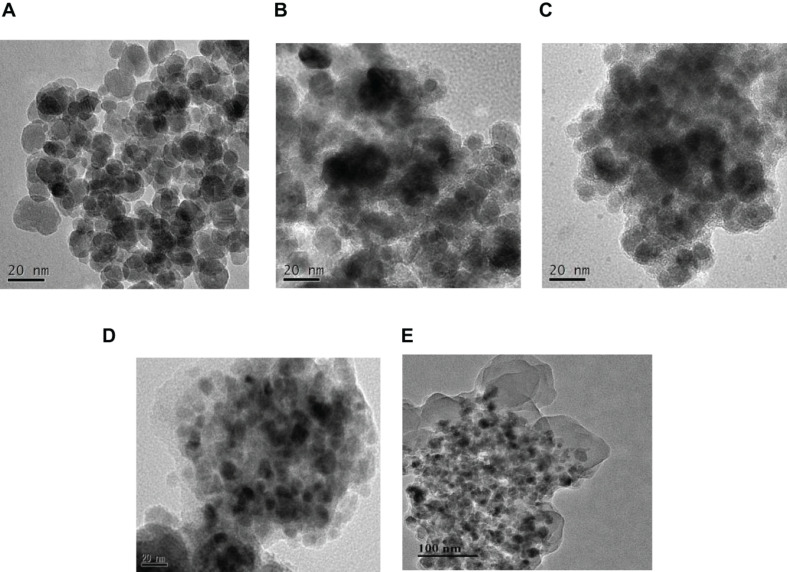
TEM image of Fe_3_O_4_ and magnetic lignin **(A)** Fe_3_O_4_, **(B)** Fe_3_O_4_@lignin (1:0.5), **(C)** Fe_3_O_4_@lignin (1:1), **(D)** Fe_3_O_4_@lignin (1:2), **(E)** Fe_3_O_4_@lignin (1:3).

The morphology and particle size distribution of the Fe_3_O_4_ and Fe_3_O_4_@lignin composites were carried out by transmission electron microscope (TEM). It can be seen from Figure 2 that the average particle size of Fe_3_O_4_ is about 20 nm, which is spherical. The particles in Fe_3_O_4_ have a strong agglomeration phenomenon. This is due to the large specific surface area of Fe_3_O_4_ nanoparticles, the surface energy is in an unstable state, and the intermolecular force, hydrogen bond, static electricity, and other forces make it agglomerate together. When Fe_3_O_4_ nanoparticles are loaded with lignin, Fe_3_O_4_ @lignin has an obvious core–shell structure, and lignin is present on the outer layer of Fe_3_O_4_. At the same time, the particle agglomeration phenomenon is suppressed to a certain extent and has a good dispersion performance. This may be due to the decrease of the surface energy of the composite magnetic particles after Fe_3_O_4_ is loaded with lignin and the reduction of the interaction force between the particles, thereby improving the dispersion performance. In addition, the particle size of Fe_3_O_4_@lignin is equivalent to that of Fe_3_O_4_, indicating that Fe_3_O_4_ modified APTES and lignin has almost no effect on the size of magnetic nanoparticles. The magnetite–lignin hybrid materials obtained by Klapiszewski et al. (2017b) also have the same aggregation tendency.

**FIGURE 3:**
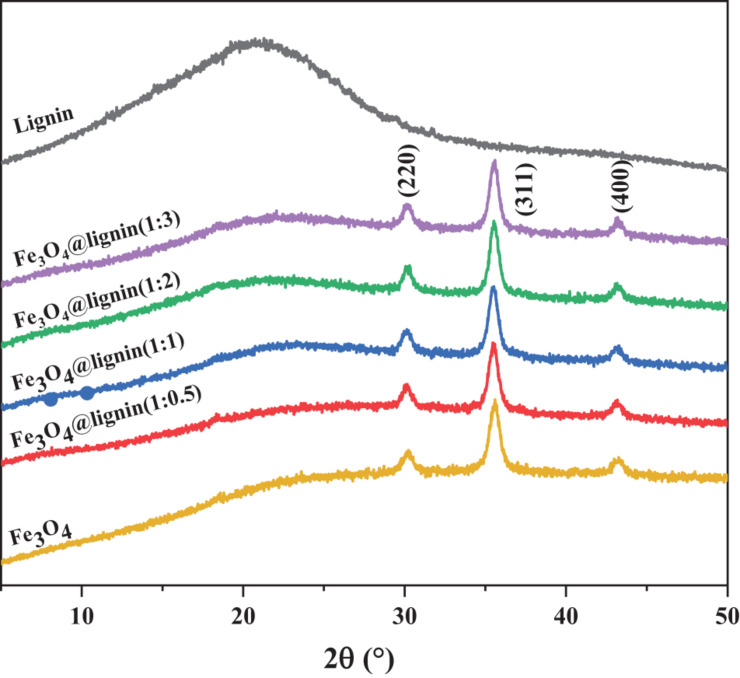
XRD spectra of magnetic lignin nanoparticles.

X-ray diffraction (XRD) was used to study the phase and crystal structure of the sample. The XRD patterns of five samples lignin and Fe_3_O_4_@lignin composites nanoparticles are shown in Figure 3. According to the literatures (An et al., 2017; Wang et al., 2019; Jia et al., 2021) that three diffraction peaks appear on the spectrum of Fe_3_O_4_ nanoparticles at 30.1°, 35.5°, and 42.9°, which were attributed to the (220), (310), and (401) crystal planes of inverse spinel Fe_3_O_4_, respectively. For lignin, only a broad diffraction peak appears around 20°, which is the diffraction peak in the amorphous region of lignin. When lignin is loaded with Fe_3_O_4_, the obtained magnetic lignin Fe_3_O_4_@lignin composites also show three crystal plane diffraction peaks of inverse spinel Fe_3_O_4_, and the position of the diffraction peaks does not shift, indicating that in the process of loading lignin to Fe_3_O_4_, it did not change its crystals.

**FIGURE 4:**
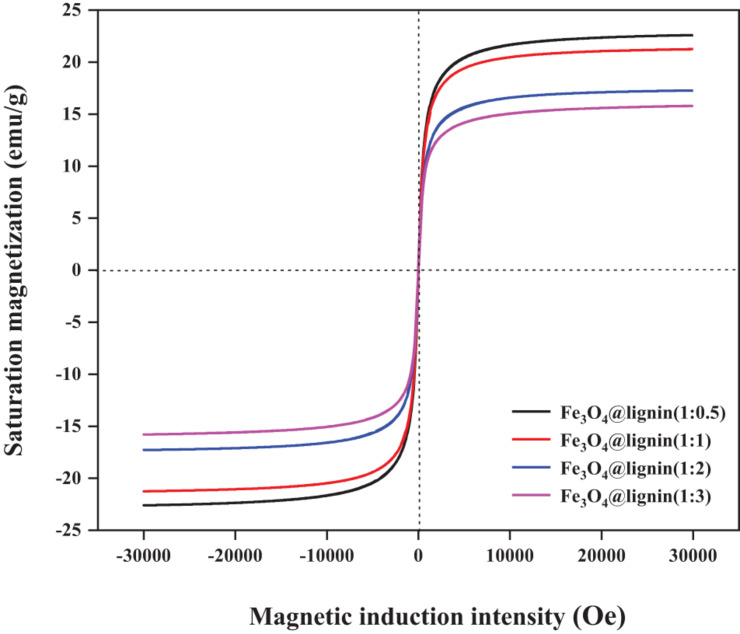
Hysteresis curve of magnetic lignin nanoparticles.

When lignin is loaded on the outer layer of Fe_3_O_4_ nanoparticles, it will not only directly affect the iron distribution of the particles, but also have a great influence on the magnetic strength of the particles (Mikhaylova et al., 2004). In addition, the magnetic strength of the magnetic adsorbent will directly affect the recovery process of the material. Therefore, the magnetic strength of Fe_3_O_4_@lignin was evaluated and shown in Figure 4. It can be seen that the hysteresis loops of the four Fe_3_O_4_@lignin composites all cross the origin and are symmetrical, which shows that the coercivity of the magnetic lignin is zero. At the same time, no remanence and hysteresis were found in these 4 hysteresis loops, indicating that the prepared magnetic Fe_3_O_4_@lignin composites all showed good superparamagnetism. Specifically, the saturation magnetization of Fe_3_O_4_@lignin with ratios of 1:0.5, 1:1, 1:2, and 1:3 was 23, 21, 16, and 15 emu g^–1^, respectively. The results indicated that the Fe_3_O_4_ loaded by a greater amount of lignin could decrease its magnetic strength. The reason for this phenomenon can be explained by the fact that the coating of lignin is a non-magnetic polymer. More coatings will increase the dipole moment of Fe_3_O_4_ particles and decrease the magnetic content, which will lead to a decrease in magnetic properties (Deatsch and Evans, 2014). In general, the results of the hysteresis curve show that the obtained Fe_3_O_4_@lignin composite material has good magnetic properties and can be easily recovered when used as an adsorbent under an external magnetic field.

**FIGURE 5:**
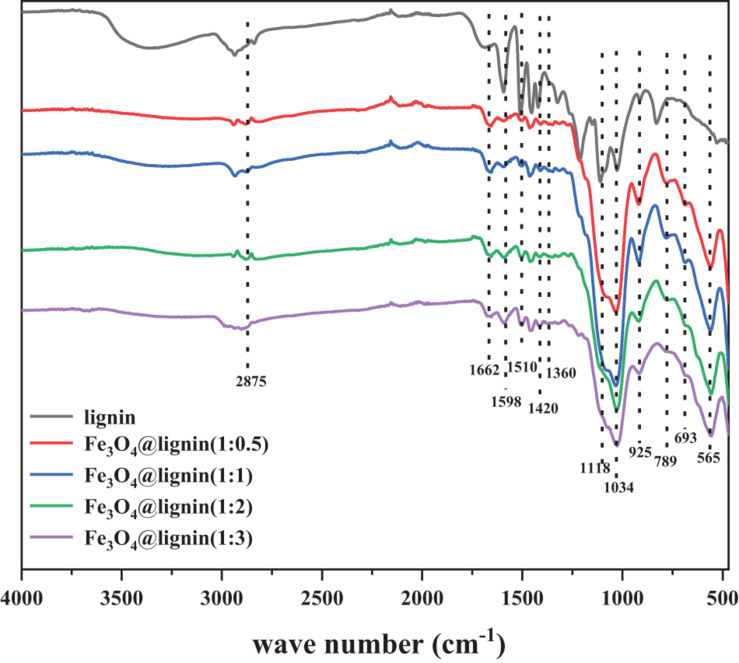
Infrared spectroscopy of magnetic lignin nanoparticles.

Infrared spectroscopy was used to analyze the structure of the prepared Fe_3_O_4_@lignin. It can be seen from Figure 5 that the lignin samples show typical signal peaks of the lignin benzene ring structure at 1,598, 1,510, and 1,420 cm^–1^ (Jiang et al., 2019). In addition, the peak at 1,360 cm^–1^ is attributable to the absorption peaks of lignin syringyl and condensed guaiacyl, and the peak at 1,118 cm^–1^ is attributable to the “S” type lignin absorption peak. The peak at 1,034 cm^–1^ indicates the vibration of the lignin H unit in the CH plane and the in-plane deformation vibration of the CH coupling bond. These lignin absorption peaks exist in the infrared spectrum of Fe_3_O_4_@lignin, indicating that the magnetic lignin obtained by the co-precipitation method will not destroy the original structure of lignin. Fe_3_O_4_ has a high-intensity absorption peak at 565 cm^–1^, which is attributed to the stretching vibration peak of Fe–O in Fe_3_O_4_ nanoparticles. The strong absorption peak was found on the absorption peak of Fe_3_O_4_@lignin, but not found in the spectrum of the lignin sample, indicating that the lignin has been successfully loaded on Fe_3_O_4_. In addition, the absorption peaks at 693, 925, and 1,662 cm^–1^ in the infrared spectrum of Fe_3_O_4_@lignin are attributed to the absorption peaks of bending vibration outside the N–H bond plane, the vibration peaks of the amino curved surface, and N–H stretching vibration peak; these phenomena indicate the successful modification of the amino group, which means that APTES has been successfully modified on Fe_3_O_4_ (An et al., 2020). Infrared analysis results show that lignin has been loaded on the surface of Fe_3_O_4_ nanoparticles to form Fe_3_O_4_@lignin.

The binding degree of lignin and iron can be determined by measuring the content of iron ions in Fe_3_O_4_@lignin. In this work, the iron content of magnetic Fe_3_O_4_@lignin is analyzed and shown in Table 1. According to Table 1, the iron content of the samples Fe_3_O_4_@lignin (1:0.5), Fe_3_O_4_@lignin (1:1), and Fe_3_O_4_@lignin (1:2) gradually increased. For Fe_3_O_4_@lignin (1:2), the iron content is the highest, and Fe_3_O_4_@lignin (1:3) is decreased. This is because the content of Fe_3_O_4_ in the prepared raw material is constant, and the maximum grafting number of Fe_3_O_4_ and lignin is fixed.

**TABLE 1:** Iron content of magnetic lignin nanoparticles.

Sample	Iron concentration (mg/g)^a^
Fe_3_O_4_@lignin (1:0.5)	2.71
Fe_3_O_4_@lignin (1:1)	3.41
Fe_3_O_4_@lignin (1:2)	5.28
Fe_3_O_4_@lignin (1:3)	3.97

*^*a*^Iron concentration (mg/g) is defined as the mass of iron ions (mg) per gram of sample.*

### Adsorption Performance of Fe_3_O_4_@lignin

**FIGURE 6:**
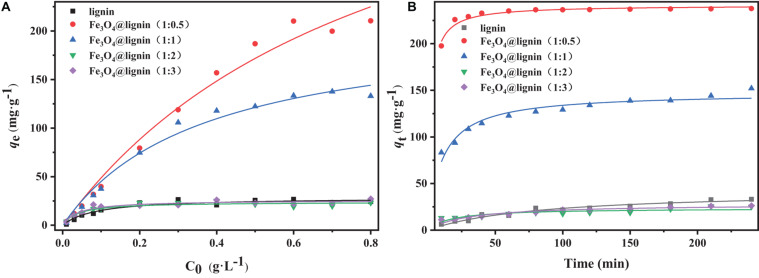
**(A)** The adsorption kinetics of lignin and Fe_3_O_4_@ lignin composites for 4 h with different concentrations of Congo red solution. **(B)** The adsorption kinetics of lignin and Fe_3_O_4_@lignin composites for Congo red with a concentration of 0.6 g/L.

The concentration of dye solution plays an important role in its absorption and decolorization percentage. The adsorption of MLN was studied by adsorbing different concentrations of Congo red dye for 4 h and the results are shown in Figure 6A. It can be seen that the adsorption amount of Congo red solution of each sample increases first with the increase of the concentration of Congo red solution. After reaching a certain concentration, it remains stable or even decreases. When the concentration was 0.6 g/L, lignin reached the maximum adsorption capacity of Congo red solution, which was 26.77 mg/g. When the concentration was 0.7g/L, Fe_3_O_4_@lignin (1:1) reached the maximum adsorption capacity of Congo red solution, which was 137.69 mg/g. When the concentration is 0.8 g/L, Fe_3_O_4_@lignin (1:0.5), Fe_3_O_4_@lignin (1:2), and Fe_3_O_4_@lignin(1:3) reached the maximum adsorption capacity of Congo red solution, which were 210.40, 23.27, and 27.13 mg/g, respectively. Among these MLN, the Fe_3_O_4_@lignin (1:0.5) showed the best adsorption capacity on Congo red and the adsorption effect is stable, which is 7.86 times that of lignin. Compared with other modified lignin or lignin-based adsorbents, the magnetic lignin obtained by cross-linking method in this study showed good adsorption properties to dyes. Wang X. et al. (2018) studied the adsorption performance of carbon composite lignin-based adsorbent for Congo red, and the maximum saturated adsorption capacity was 198.5 mg/g. Jiang et al. (2019) studied the adsorption performance of magnetic lignin adsorbent (Fe_3_O_4_/C-ACLS) for Congo red, and the maximum saturated adsorption capacity was 198.8 mg/g.

The adsorption performance of magnetic lignin for Congo red during different adsorption times has a great significance for its industrial utilization in dye adsorbing. Hence, the lignin and Fe_3_O_4_@lignin composites were investigated for adsorption of Congo red at a concentration of 0.6 g/L within 0–300 min, as shown in Figure 6B. It can be seen that the adsorption effect of Fe_3_O_4_@lignin (1:0.5) on Congo red reaches saturation at 30 min and the adsorption capacity is as high as 229.1 mg/g, and the adsorption effect remains stable after 30 min. For the other MLN, their adsorption capacity also increased steadily with the increase of time and reached the maximum adsorption capacity at 240 min. Specifically, lignin, Fe_3_O_4_@lignin (1:1), Fe_3_O_4_@lignin (1:2), and Fe_3_O_4_@lignin (1:3) reached the maximum adsorption capacity of 33.10, 152.13, 25.22, and 26.23 mg/g, respectively. Wang et al. (2014) studied the ultrasonic-assisted synthesis of aminated lignin by a Mannich reaction as an adsorbent to adsorb Congo red with yield of 95.5% at 48 h. Hence, it can be speculated that the MLN of Fe_3_O_4_@lignin (1:0.5) can be regarded as the best adsorber to treat dye wastewater due to its largest adsorption capacity and fastest adsorption rate.

Based on the analysis of the initial concentration and adsorption time, it can be found that Fe_3_O_4_@lignin (1:0.5) is superior than other MLN to adsorb Congo red. Moreover, the adsorption capacity of 0.6 g/L Congo red solution by Fe_3_O_4_@lignin (1:0.5) for 30 min is the equilibrium adsorption amount, which is the focus of this study.

### Adsorption Kinetics

**FIGURE 7:**
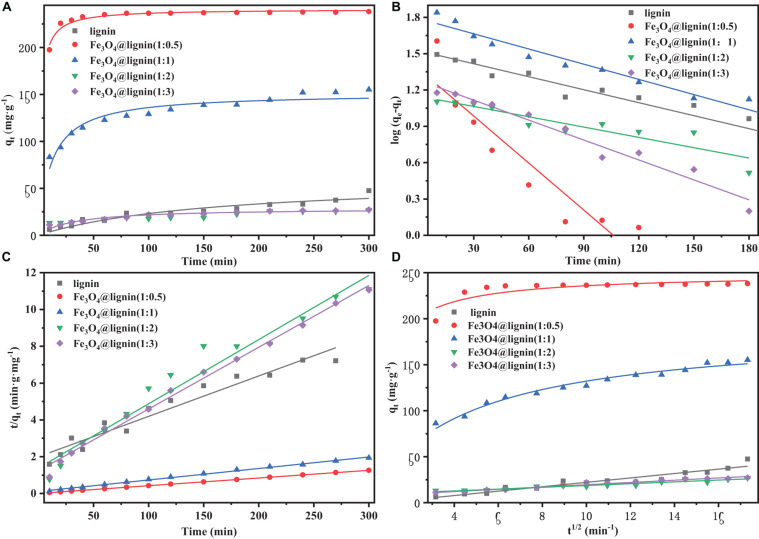
**(A)** Effects of different adsorbents on Congo red adsorption at different times. **(B)** The pseudo-first-order kinetics model curve examined. **(C)** The pseudo-second-order kinetics model curve examined. **(D)** The intraparticle diffusion model.

Adsorption isotherms play an important role in understanding the adsorption mechanism for different adsorbent. In this study, different concentrations of dyes were used to investigate the adsorption mechanism of prepared MLN, which is shown in Figure 7.

The pseudo-first-order kinetic equation assumes that the adsorption process is controlled by diffusion. The pseudo-second-order kinetic equation assumes that the adsorption rate is controlled by a chemical adsorption mechanism (Nair et al., 2014). This chemisorption involves electron transfer or electron pairing between the adsorbent molecules and the adsorbates. For the intraparticle diffusion equation, if there is intraparticle diffusion, q_*t*_ has a linear relationship with t^0.5^, and if the straight line passes through the origin, the rate control process is only controlled by the internal diffusion. Otherwise, other adsorption mechanisms will be accompanied by internal diffusion process at the same time.

Figure 7B and Table 2 show the pseudo-first-order curve fitting model and the model parameters, respectively. The R^2^ values for each plot are between 0.49 and 0.99. Figure 7C and Table 2 show the pseudo-second-order curve fitting model and model parameters. The R^2^ values for each plot are between 0.64 and 0.99, which is higher than the holistic coefficient value (R^2^) of the pseudo-first-order kinetic equation. These results showed that the adsorption process of MLN in Congo red is in good agreement with the pseudo-second-order model; the chemical adsorption plays a leading role for MLN comparing with diffusion.

**TABLE 2:** Kinetic parameters for the adsorption of Congo red dye on lignin and magnetic lignin composites.

Kinetics model	Parameter	Lignin	Fe_3_O_4_@lignin			
			
			1:0.5	1:1	1:2	1:3
Pseudo-first order	*q*_*e exp*_ (mg/g)	37.43	237.69	152.44	25.78	26.73
	*q*_*e cal*_ (mg/g)	34.52	236.60	136.43	21.14	24.66
	*k*_1_ (× 10^–2^/min)	0.10	17.90	6.30	4.10	2.40
	*R*^2^	0.89	0.99	0.68	0.49	0.83
Pseudo-second order	*q*_*e exp*_ (mg/g)	37.43	237.69	152.44	25.78	26.73
	*q*_*t cal*_ (mg/g)	32.67	261.13	148.27	22.26	28.21
	*k*_2_ (× 10^–2^ g/mg min^–1^)	0.06	0.12	0.07	0.33	0.11
	*R*^2^	0.93	0.98	1.00	0.64	0.99
Intraparticle diffusion model	*k*_*i*_ (mg/g min^1/2^)	2.45	1.57	4.59	1.04	1.21
	*C* (mg/g)	1.90	216.00	80.18	8.48	7.50
	*R*^2^	0.94	0.46	0.94	0.94	0.97

The curves in Figure 7D showed that the values between q_*t*_ and t^0.5^ signifying intra-particle diffusion model is fitting for the adsorbing, where the value of “C” corresponding to the intercept helped to explain whether the control step is intra-particle diffusion or not. According to the data in Table 2, C was a non-zero value which indicated that the relationship between t^0.5^ and q_*t*_ is not a straight line passing through the origin, showing that the adsorption rate is controlled by multiple adsorption mechanisms. Two or more steps occur in the process; adsorption process also includes complex mechanism pathways (Sohni et al., 2018). Jiang et al. (2019) showed that the adsorption rate was generally controlled by three stages. The first stage is the initial stage of the reaction, which is the diffusion stage of the dye in the boundary layer, where the adsorption capacity increases faster. As the reaction continues, the adsorption capacity in the second stage still increases, but the increase rate slows down. The reaction process in this stage is controlled by intraparticle diffusion. In the third stage, the adsorption reaches equilibrium. It can be seen from Figure 7D that the adsorption rate of Fe_3_O_4_@lignin (1:0.5) is faster, and the figure shows the three stages of the adsorption process. While the adsorption rate of the other samples is slower, the figure only shows the second step between the initial rapid external diffusion stage and the equilibrium stage in the adsorption process. The adsorption rate of the second step is relatively stable to each sample, so the second step is chosen to characterize the rate parameters corresponding to diffusion.

The calculated k_*i*_ values for each initial concentration are shown in Table 2. It can be seen that the R^2^ values of the lignin and Fe_3_O_4_@lignin (1:1) (1:2) (1:3) for the diffusion model were all above 0.9. This indicated that the adsorption process could be followed by an intra-particle diffusion after around 10 min (Liu L. et al., 2018). In contrast, the R^2^ value of the adsorption curve for Fe_3_O_4_ @lignin (1:0.5) presented a really low R^2^ value of 0.46. This may be due to the adsorption capacity of the adsorbent Fe_3_O_4_@lignin (1:0.5) for Congo red that is too strong and the high adsorption rate of adsorbent, resulting in the disorder of adsorption curve, which is also consistent with the conclusion of Figure 7D. Overall, from the aforementioned results, it can be concluded that the pseudo second-order model is the best model to describe the adsorption kinetics of MLN for adsorbing the Congo red dye.

### Adsorption Isotherm

The adsorption isotherm refers to the relationship between the mass of the dye adsorbed on the unit mass of the adsorbent and the concentration of the liquid dye under constant temperature conditions (Masilompane et al., 2018). The equilibrium data were analyzed based on the Langmuir adsorption isotherm and the Freundlich adsorption isotherm. Among them, the Langmuir adsorption isotherm assumes that the adsorbate adsorbs on the monolayer of the surface of the homogeneous adsorbent, and the adsorption activation energy of each molecule adsorbed on its surface is equal. Adsorption migration did not occur after Langmuir adsorption isotherm adsorption. The Freundlich adsorption isotherm describes reversible adsorption not limited to the formation of a single layer; the adsorption is an interaction between heterogeneous multilayer molecules and adsorbates, and the adsorption capacity increased with the increase of the concentration.

From the correlation coefficient (R^2^) in Table 3, it can be seen that Fe_3_O_4_@lignin (1:0.5) Freundlich isotherm model R^2^ is closer to 1 than the Langmuir isotherm model R^2^. The Freundlich isotherm R^2^ for Fe_3_O_4_@lignin (1:1) is consistent with the Langmuir model R^2^. Other adsorbents perform better on Langmuir isotherms than Freundlich isotherms, indicating that except for Fe_3_O_4_@lignin (1:0.5), (1:1), the process of adsorbing Congo red is multilayer adsorption, and the adsorption processes of other adsorbents are all monolayer adsorption. What is more, the value of the separation factor R_*L*_ is greater than 0 and less than 1, which results demonstrated that the adsorption process is favorable. This result is consistent with the results of Li et al. (2019) that showed the adsorption of Congo red is more consistent with the Langmuir model, indicating that the adsorption process of Congo red is monolayer adsorption.

**TABLE 3:** Adsorption isotherm model parameters.

Parameters	Lignin	Fe_3_O_4_@lignin			
		
		1:0.5	1:1	1:2	1:3
**Langmuir**					
*q*_*m*_ (mg/g)	28.72	470.02	206.29	25.30	27.10
*b* (× 10^–2^ L/mg)	1.10	0.11	0.28	2.27	2.33
*R*^2^	0.96	0.98	0.98	0.97	0.93
*R*_*L*_ = 1/(1+bC_0_)	0.90	0.98	0.97	0.78	0.81
**Freundlich**					
*K*_*f*_ (L/g)	2.28	0.42	0.74	1.97	1.99
*1/n*	0.41	0.98	0.95	0.44	0.44
*R*^2^	0.80	1.00	0.98	0.89	0.82

The value of theoretical maximum of adsorption capacity (q_*m*_) of the lignin and MLN of Fe_3_O_4_@lignin (1:0.5), Fe_3_O_4_@lignin (1:1), Fe_3_O_4_@lignin (1:2), and Fe_3_O_4_@lignin (1:3) derived from the Langmuir model were 28.72, 470.02, 206.29, 25.30, and 27.10 mg/g, respectively, of which lignin, Fe_3_O_4_ @lignin(1:2), and Fe_3_O_4_ @lignin (1:3) were consistent with the corresponding experimental values. The q_*m*_ values of Fe_3_O_4_ @lignin (1:0.5) and Fe_3_O_4_ @lignin (1:1) were higher than the experimental data, indicating that these two samples do not conform to the Langmuir model, which is consistent with previous conclusions. According to the Freundlich model, 1/n values of lignin, Fe_3_O_4_@lignin (1:2), and Fe_3_O_4_@lignin (1:3) are greater than 0 and less than 1, showing that the adsorption is advantageous. 1/n values of Fe_3_O_4_@lignin (1:0.5) and Fe_3_O_4_@lignin (1:1) are close to 1, showing that the adsorption is linear and there is no interaction between adsorbates. The reason for this phenomenon might be due to the high adsorption efficiency of Fe_3_O_4_@lignin (1:0.5) and Fe_3_O_4_@lignin (1:1), which leads to inaccurate adsorption model.

## Conclusion

Studies have shown that MLN is an effective Congo red adsorbent with good adsorption performance, simple preparation, and easy recovery, which is an ideal adsorbent for Congo red dye. For the prepared MLN with different ratios of Fe_3_O_4_@lignin, Fe_3_O_4_@lignin (1:0.5) showed the best adsorption performance on Congo red with adsorption rate of 95.5% and adsorbing ability of 229 mg/g. The kinetics and isothermal models show that adsorption process of Fe_3_O_4_@lignin (1:0.5) belonged to the chemical adsorption of the multimolecular layer, and the adsorption process of other adsorbents is in accordance with the chemical adsorption of the monomolecular layer.

## Data Availability Statement

The raw data supporting the conclusions of this article will be made available by the authors, without undue reservation.

## Author Contributions

LF: investigation. HW and YT: supervision. YS: writing. QY: writing—review and editing. All authors contributed to the article and approved the submitted version.

## Conflict of Interest

The authors declare that the research was conducted in the absence of any commercial or financial relationships that could be construed as a potential conflict of interest.
